# A role for the microbiota in complex regional pain syndrome?

**DOI:** 10.1016/j.ynpai.2020.100054

**Published:** 2020-11-25

**Authors:** Lara W. Crock, Megan T. Baldridge

**Affiliations:** a Department of Anesthesiology and Pain Medicine, Washington University in St. Louis, St. Louis, MO, USA; b Division of Infectious Diseases, Department of Medicine, Washington University in St. Louis, St. Louis, MO, USA

**Keywords:** Complex regional pain syndrome, Gut microbiome, Gut microbiota, Chronic pain, Metabolomics

## Abstract

Complex regional pain syndrome (CRPS) is a debilitating neuroinflammatory condition of unknown etiology. Symptoms include excruciating pain and trophic changes in the limbs as defined by the Budapest criteria. The severity and functional recovery of CRPS, unlike most pain conditions, is quantifiable using a variation of the Budapest criteria known as the CRPS severity score. Like many chronic pain conditions, CRPS is difficult to treat once pain has been present for more than 12 months. However, previous work has demonstrated that a subset of patients with new-onset CRPS (~50%) improve if treated within one year, while the rest have minimal to no symptom improvement. Unfortunately, this leads to permanent disability and often requires invasive and costly treatments such as spinal cord stimulation or long-term opioid therapy. Because the etiology is unknown, treatment is multimodal, and often supportive. Biomarkers that predict severity or resolution of symptoms would significantly change treatment but have not yet been identified. Interestingly, there are case reports of remission or resolution of CRPS symptoms with the use of antibiotics known to affect the gut flora. Mouse studies have demonstrated that modulation of the gut microbiome is anti-nociceptive in visceral, inflammatory and neuropathic pain models. We hypothesize that the variable clinical potential for recovery and response to therapy in CRPS may be secondary to or reflected in changes in the gut microbiota. We suggest that the microbiota may mediate or reflect clinical status via the metabolome, activation of the immune system and/or microglial activation. We hypothesize that the gut microbiome is a potential mediator in development and persistence of CRPS symptoms and propose that the clinical condition of CRPS could provide a unique opportunity to identify biomarkers of the microbiota and potential therapies to prevent pain chronification.

## Highlights

• Complex Regional Pain Syndrome (CRPS), a rare, painful condition of unknown etiology.
• Once present for more than one year, CRPS is difficult to treat.
• Preclinical research has demonstrated a role of gut microbiota in the development of pain.
• The Gut microbiota may modulate CRPS via modulate the acute metabolites, the immune system and/or microglia.

## 1 Introduction

Complex regional pain syndrome (CRPS), formally known as reflex sympathetic dystrophy (RSD) or causalgia, is a devastating, persistent neuropathic pain condition that occurs most often following stressful events such as surgery or trauma (Harden et al., 2010, Blaes et al., 2015). The development of CRPS remains a controversial enigma. Often, peripheral limb trauma leads to an exaggerated inflammatory response, causing a constellation of symptoms such as redness, swelling and increased temperature that are often initially mistaken for infection. As pain persists, trophic changes and central sensitization occur, potentially via the production of autoantibodies, neuroinflammation and/or local release of cytokines (Linnman et al., 2013, Birklein et al., 2018). CRPS is traditionally considered acute if the symptoms started within 12 months, and chronic thereafter.

**Fig. 1:**
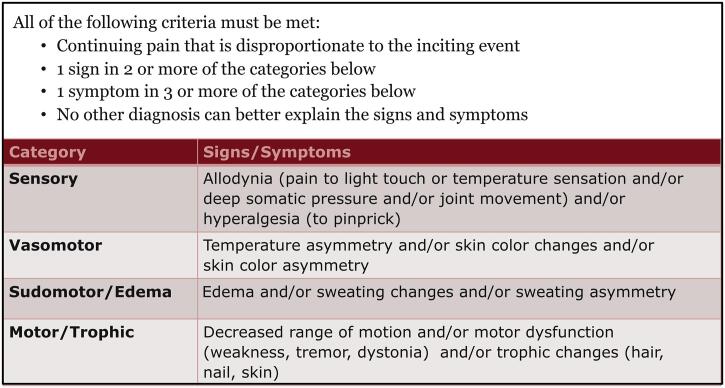
The Budapest Criteria. Complex Regional Pain Syndrome (CRPS) is a diagnosis of exclusion. The accepted criteria for the diagnosis of CRPS is based on observed signs in two or more categories and reported symptoms in three or more of the listed categories (Harden et al., 2010).

Type I CRPS occurs in the absence of major nerve trauma, while type II occurs in the setting of known nerve trauma. Both types of CRPS are diagnoses of exclusion and require pain that is out of proportion to the initial injury as well as signs in at least two and symptoms in at least three of the following categories: sensory, vasomotor, sudomotor and motor/trophic (the Budapest Criteria, Fig. 1) (Harden et al., 2010). CRPS severity and recovery is quantifiable using a composition of the signs and symptoms, the validated CRPS severity score (Harden et al., 2017). The ability to quantify the severity of a pain condition allows for more rigorous and reliable outcome measures. Development of CRPS is life-altering. Patients report decreased mobility, inability to work, poor quality of life, depression, anxiety, and often long-term opioid usage (Birklein et al., 2018). We do not yet understand why some patients will develop CRPS following an injury, or what factors may explain why some recover while others don’t.

## 2 Treatment of CRPS

Treatment options for CRPS include conservative therapies such as medications, physical therapy, and/or injections (O'Connell et al., 2016). Although highly debated, early symptoms of CRPS are largely thought to be sympathetically mediated, which results in the use of sympathetic blocks for symptom management (Birklein and Dimova, 2017). Although commonly performed, robust evidence for their use of is poor (O'Connell et al., 2016). Experimental therapies such as the use of intravenous immunoglobulin have been disappointing (Goebel et al., 2014). Sadly, 20–50% of patients with less than 12 months of symptoms fail conservative management and may progress to chronic opioid therapy or implantation of a neuromodulation device such as a spinal cord or dorsal root ganglion (DRG) stimulator (Birklein et al., 2015, Mekhail et al., 2020). DRG stimulators have promise in early studies but can only be used in CRPS of the lower extremity with specific anatomical considerations. Even with the newest spinal cord stimulator therapies, only about 50% of patients will have a successful response, which is defined as at least 50% pain relief. Treatment efficacy decreases further when the disease becomes chronic (O'Connell et al., 2013).

## 3 CRPS prognosis

In the absence of significant confounders such as concomitant psychological or neurocognitive disease, greater than 50% of patients with CRPS experience significant functional and symptomatic recovery (Birklein et al., 2015). Unfortunately, recovery most commonly occurs early in the disease, and is uncommon after a year or more of symptoms.

The difference in potential for recovery may be due to inherent molecular differences between early and chronic CRPS. Similarly, it is believed that distinct, as-of-yet undescribed, subtypes of CRPS may exist (Harden et al., 2010). Local cytokine levels of interleukin-1 receptor antagonist (IL-1RA), IL-6, TNF-alpha, monocyte chemotactic protein-1 (MCP-1) and macrophage inflammatory protein-1beta (MIP-1beta) are higher in induced-blister fluid from the affected limb of patients with early (less than 1 year duration) CRPS when compared to the unaffected limb (Heijmans-Antonissen et al., 2006, Lenz et al., 2013). However, these biomarkers have no predictive value when CRPS is present for more than one year (chronic CRPS) (Lenz et al., 2013). To date, we do not have biomarkers to predict which patients are at risk to develop or have the potential to recover from CRPS (Birklein et al., 2018). Identifying factors that contribute to CRPS recovery are critical to potentially preventing and/or curing this debilitating condition. We propose that the heterogeneity of CRPS symptoms and response to treatment may be related to microbiota differences.

## 4 Microbiota and pain

There has been a recent surge of interest in the potential role of the gut microbiota in pain, detailed in several excellent reviews (Dworsky-Fried et al., 2020, Guo et al., 2020). Preclinical work in mouse models has shown that the presence of intact gut microbiota is necessary for the development of normal visceral pain sensation (Luczynski et al., 2017); and abnormal gut microbiota composition can contribute to the development of irritable bowel syndrome as well as IBS, neuropathic and inflammatory pain (Ramakrishna et al., 2019, Shen et al., 2017, Yang et al., 2019). Clinical studies have uncovered decreased bacterial diversity or dysbiosis of the human gut microbiome associated with various painful conditions such as visceral pain (Guo et al., 2017), fibromyalgia (Clos-Garcia et al., 2019), and arthritic knee pain (Boer et al., 2019). Decreased gut bacterial diversity has also been observed in conditions with significant overlap with chronic pain such as major depressive disorder (Jiang et al., 2015). Prebiotics known to cause changes to the gut microbiome have been shown to be therapeutic in clinical scenarios including improved mood (Kazemi et al., 2019). Furthermore, a double-blind randomized, placebo-controlled study showed a synergistic effect of the probiotic *Lactobacillus plantarum 299v* with a selective serotonin reuptake inhibitor for improved cognitive function in patients suffering from major depression (Rudzki et al., 2019). *Bifidobacteria* and *Lactobacillus* probiotics have been beneficial in treating the abdominal pain of irritable bowel syndrome (Bonfrate et al., 2020). However, no clinical studies have evaluated the treatment of neuropathic pain. Interestingly, there are several case reports of patients with CRPS recovering after treatment with antibiotics that are known to alter the microbiome (Ware and Bennett, 2014, Weinstock et al., 2016). In both reports, the patients suffered from CRPS for an extended period of time before they were placed on antibiotics for an unrelated condition. These reports raise the intriguing possibility of a link between the microbiota and chronic maintenance of CRPS.

## 5 Metabolites and pain

Microbes metabolize resistant starches and dietary fibers through decomposition and fermentation and thus provide short chain fatty acids (SCFAs, including formic, pyruvic, butyric, lactic and acetic acids) to the host, in addition to processing bile acids and other metabolites. Gut bacteria are thought to be the main producers of SCFAs, which have been shown to modulate inflammation through leukocyte recruitment, chemokine production (Vinolo et al., 2011), regulation of tight junctions in the intestinal wall and integrity of the blood–brain barrier (BBB) (Braniste et al., 2014, Yamamoto and Jorgensen, 2019). The composition of the gut microbiome has also been shown to play an important role in microglial homeostasis through microbiota-derived fermentation products, production of SCFAs, and modulation of the immune system (Lyte, 2014, Fung et al., 2017).

Metabolites, specifically SCFAs, have been recently shown to play a role in the progression of painful conditions such as rheumatoid arthritis (RA) through a B-cell dependent mechanism (Rosser et al., 2020). Stool from patients with RA exhibit reduced butyrate levels when compared to healthy controls. In a mouse RA model, a similar reduction in stool butyrate was found. Furthermore, oral supplementation with butyrate is protective in a preclinical model of arthritis in a B-cell dependent mechanism. Suppression of disease is associated with a reduction in TNF-alpha, IL-6 and MCP-1 produced by lymphocytes in the lymph node draining the affected limb. However, this study was limited by outcome measures; arthritis severity was measured by swelling alone and did not include pain behaviors (Rosser et al., 2020). In a rat neuropathic preclinical model, oral butyrate is anti-nociceptive (Kukkar et al., 2014). Furthermore, circulating levels of butyrate may be one of the mechanisms of decreased mechanical hypersensitivity as the result of fecal transplantation from lean mice into obese, insulin-resistant mice (model of diabetic peripheral neuropathic pain) (Bonomo et al., 2020).

Network modelling and pathway mapping tools can help identify the roles that metabolites play in relation to each other and in biological aberrations. The identification of important metabolomic biomarkers for pain in conjunction with taxonomic biomarkers can lead to mechanistic insights and proof of concept with manipulation of these pathways (Johnson et al., 2016). In a cohort of patients suffering from fibromyalgia, serum metabolomics were correlated with the observed reduction in gut microbiome diversity (Clos-Garcia et al., 2019). In this study, decreased abundance of *Bifidobacterium, Lactobacillus* and *Eubacterium* genera (bacteria known to produce SCFAs) was associated with reduced glutamate and serine in the serum. This study proposed that the differences in microbiota might cause the observed reduction in serum glutamate, and potentially increase pain through glutamatergic synapses (Clos-Garcia et al., 2019). The addition of gut metabolomic analysis in future studies would additionally help to clarify these connections. Understanding the composition of the gut microbiota in patients with CRPS, as well as potential fecal and serum metabolites changes, will help elucidate if differences exist between patients with a recent diagnosis of CRPS (acute) compared to patients who have recovered or have persistent symptoms (chronic). Such differences could suggest a possible mechanism of action for CRPS pain chronification.

## 6 Microglia and CRPS

**Fig. 2:**
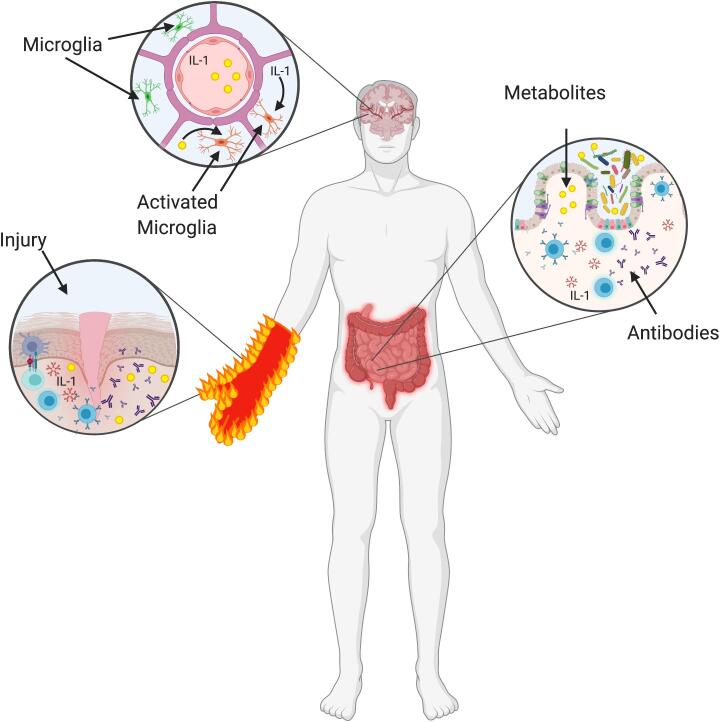
Potential Mechanisms of CRPS. Several hypothesized mechanisms of the development and persistence of Complex Regional Pain Syndrome (CRPS). Microbiota in the gut prime the immune system to produce antibodies (including auto-antibodies) and release metabolites (such as short chain fatty acids) and cytokines (for example, IL-1). After a peripheral injury, local auto-antibodies and cytokines are released to initiate an inappropriate inflammatory response resulting in severe pain. Microbiota-produced metabolites and/or cytokines may cross the blood brain barrier to result in microglial activation and thus pain chronification. Created with BioRender.com.

One potential mechanism of the gut microbiome’s influence on persistent pain is through modulation of microglial reactivity (Dworsky-Fried et al., 2020, Ramakrishna et al., 2019, Haight et al., 2019). Microglia, non-neuronal cells in the brain, make up 10% of the total central nervous system (CNS) cell population and their functions include initiation of neuronal apoptosis, clearing dead cells and the pruning/re-modeling of synapses. These functions are modulated by other glial cells, neurons as well as exogenous factors such as SCFAs produced by the gut microbiota (Erny and Prinz, 2020). Germ-free mice have developmentally immature microglia (Erny et al., 2015), suggesting that the presence of an intact gut microbiome is necessary for the functional maturation of microglia in the CNS. Manipulation of the gut microbiota in mice alters extinction learning through microglia-mediated synaptic pruning (Chu et al., 2019). Microglial activation has been implicated in the development of chronic pain (Haight et al., 2019), CRPS (Littlejohn, 2015) and preclinical models of CRPS (Helyes et al., 2019). One of the most studied microglial modulators is minocycline, a tetracycline antibiotic. In many, but not all, preclinical models, the administration of minocycline prevents microglial activation and reverses injury-induced allodynia and hyperalgesia. However, clinical trials of minocycline have been disappointing (excellent summary in (Haight et al., 2019)). The failure of minocycline as a pain preventative may not be just the results of off target effects but perhaps due to the inherent complexity of the human gut microbiota in comparison to that of mice (Nguyen et al., 2015). The mechanisms by which microglia modulate the persistence of pain are unknown, and the neuromodulatory signals from the gut microbiota are one potential mediator (Fig. 2).

## 7 The Microbiota, inflammation and CRPS

Our immune system learns appropriate and inappropriate responses partially through interactions with symbiotic bacteria. Here we will briefly discuss how the microbiota may be involved in aberrant local and systemic immune responses in CRPS (Fig. 2). Inflammation, in controlled settings, is important for appropriate recovery from injury. The composition of the intestinal microbiota is important for the ability to mount a local immune response through antibody production (Cisalpino et al., 2017, Yamamoto and Jorgensen, 2019), and also for the initiation of a local inflammatory response to injury. Germ-free and flora-depleted mice are protected against the effects of ischemia and reperfusion injury of the intestine (Cisalpino et al., 2017), brain (Benakis et al., 2016) and kidney (Emal et al., 2017) because they do not mount an appropriate inflammatory response. However, serum transferred from conventional mice into germ-free mice is sufficient to rescue the inflammatory response to an ischemic bowel injury, suggesting that factor(s) in the serum of conventional mice are necessary for these responses (Cisalpino et al., 2017). Although controversial, multiple studies have implicated the local immune system and/or inappropriate post-traumatic inflammation in the development and persistence of CRPS. The changes that occur in CRPS are localized in both the observed phenotype (unilateral limb affected) as well as the lack of increased systemic inflammatory markers. In patients suffering from acute CRPS, only the inflammatory cytokines in blister fluid of the affected limb, and not total serum cytokine levels, are increased when compared to patients with similar injuries without CRPS (Kramer et al., 2011, Lenz et al., 2013).

The microbiota may also contribute to the development of autoimmune disorders through production of autoantibodies (IgM or IgG). This is thought to occur through “molecular mimicry” where microbial peptides that are structurally similar to self-antigens initiate auto-reactivity. The gut microbiota’s role in autoantibody production is an active area of research in several autoimmune disorders such as multiple sclerosis, systemic lupus erythematosus and type 1 diabetes (Yamamoto and Jorgensen, 2019). Preclinical evidence supports a role for IgG and/or IgM antibodies in CRPS. Mice that undergo a small skin-muscle incision developed CRPS-like changes in their paw after treatment with serum IgG from patients with CRPS (Helyes et al., 2019). Hyperalgesia and swelling of the paw were accompanied by activation of microglia and astrocytes in the spinal cord dorsal horn. These changes were attenuated by pharmacologic blockade or genetic deletion of interleukin-1 (IL-1, Fig. 2). The role of IgG in CRPS was supported in a small cohort (20 subjects) where 55–90% of patients with CRPS had anti-autonomic IgG autoantibodies in their serum (Kohr et al., 2011, Dubuis et al., 2014). This ultimately led to the therapeutic use of plasmapheresis and the use of intravenous immunoglobulin in clinical trials of CRPS. Although initially promising in case reports (Dubuis et al., 2014, Blaes et al., 2015), larger studies were not efficacious (Goebel et al., 2014).

The microbiota plays broad roles in both humoral immunity (B cell development and proinflammatory T cell responses) as well as immune regulation (regulatory B and T cells). Recent preclinical models of CRPS have also demonstrated an important role of B cells and IgM in the development of pain and swelling following distal tibial fracture and casting injury (Guo et al., 2017). Manipulation of the immune system through B-cells either by anti-CD-20 antibodies (rituximab) or a genetic B-cell deficiency (muMT) resulted in reduced pain and swelling in a preclinical CRPS-like model (Li et al., 2014). Furthermore, transfer of serum or IgM complexes from injured wild-type mice into mice with a B cell deficiency rescued the pain behaviors in a time frame consistent with half-life of IgM (Guo et al., 2017). In this model, systemic injection of IgM antibodies (but not IgG antibodies) from patients suffering from CRPS was sufficient to rescue the pain behaviors of injured mice lacking B cells (Guo et al., 2020). Although purely theoretical, it is possible that the gut microbiota functions to prime the immune system in CRPS resulting in an exaggerated response to an injured limb.

## 8 Future perspectives and challenges

Here, we provide a review of the latest scientific advancements in CRPS research and propose a potential role for the gut microbiota as a potential biomarker for diagnosis, treatment and clinical course prediction. Current preclinical and clinical data support a highly testable role of the microbiota as a potential risk factor for the development and persistence of CRPS. We hypothesize that CRPS is an ideal condition to elucidate this role as the severity is quantifiable and many patients will recover. The evaluation of the clinical severity of CRPS (e.g. CRPS severity score) in conjunction with fecal taxonomic and metabolomic analysis of patients with acute CRPS through either their recovery or persistence of pain may identify biomarkers of the acute to chronic pain transition. Furthermore, these data may help us understand potential mechanisms and targeted treatments to facilitate patient recovery. Because this is a well-characterized and quantifiable pain condition, we propose that CRPS is an intriguing clinical area to assess the microbiota’s role in the development of chronic pain.

## CRediT authorship contribution statement

**Lara W. Crock:** Conceptualization, Writing - original draft, Writing - review & editing, Data curation. **Megan Baldridge:** Writing - review & editing.

## Declaration of Competing Interest

The authors declare that they have no known competing financial interests or personal relationships that could have appeared to influence the work reported in this paper.
